# Performance Study of F-P Pressure Sensor Based on Three-Wavelength Demodulation: High-Temperature, High-Pressure, and High-Dynamic Measurements

**DOI:** 10.3390/s24165313

**Published:** 2024-08-16

**Authors:** Maocheng Guo, Qi Zhang, Hongtian Zhu, Rui Liang, Yongqiu Zheng, Xiang Zhu, Enbo Wang, Zhaoyi Li, Chenyang Xue, Zhenyin Hai

**Affiliations:** 1 School of Aerospace Engineering, Xiamen University, Xiamen 361102, China; 19920221151622@stu.xmu.edu.cn (M.G.); 19920231151668@stu.xmu.edu.cn (Q.Z.); 35020230156555@stu.xmu.edu.cn (H.Z.); wangenbo2002@foxmail.com (E.W.); lizhaoyizhenxing@163.com (Z.L.); 2 State Key Laboratory of Dynamic Measurement Technology, North University of China, Taiyuan 030051, China; s202206095@st.nuc.edu.cn (R.L.); zhengyongqiu@nuc.edu.cn (Y.Z.); s202306140@st.nuc.edu.cn (X.Z.)

**Keywords:** dynamic signals, demodulation algorithm, high temperature and high pressure, Fabry–Perot sensor

## Abstract

F-P (Fabry–Perot) pressure sensors have a wide range of potential applications in high-temperature, high-pressure, and high-dynamic environments. However, existing demodulation methods commonly rely on spectrometers, which limits their application to high-frequency pressure signal acquisition. To solve this problem, this study developed a self-compensated, three-wavelength demodulation system composite with an F-P pressure sensor and a thermocouple to construct a comprehensive sensing system. The system produces accurate pressure measurements in high-temperature, high-pressure, and high-dynamic environments. In static testing at room temperature, the sensing system shows excellent linearity, and the pressure sensitivity is 158.48 nm/MPa. In high-temperature testing, the sensing system maintains high linearity in the range of 100 °C to 700 °C, with a maximum pressure-indication error of about 0.13 MPa (0~5 MPa). In dynamic testing, the sensor exhibits good response characteristics at 1000 Hz and 5000 Hz sinusoidal pressure frequencies, with a signal-to-noise ratio (SNR) greater than 37 dB and 45 dB, respectively. These results indicate that the sensing system proposed in this study has significant competitive advantages in the field of high-temperature, high-speed, and high-precision pressure measurements and provides an important experimental basis and theoretical support for technological progress in related fields.

## 1. Introduction

Pressure measurement in high-temperature environments has garnered significant interest [1]. While conventional electrical-signal pressure sensors exhibit good performance at normal temperatures, they are constrained to measurements at 500 °C due to the propensity of silicon-based sensitive materials to undergo plastic deformation at elevated temperatures [2]. Pressure sensors utilizing SiC materials offer advantages at 600 °C; however, their high-temperature applications are limited by temperature resistance due to the silicon diaphragm and underlying electrodes [3].

Fiber optic F-P sensors are characterized by high-temperature resistance, compact size, resistance to electromagnetic interference, and high sensitivity. They exhibit a wide range of potential applications in aerospace, turbine engines, oil, gas, and other highly dynamic measurement technologies [4,5,6]. In dynamic testing, F-P sensors have been extensively used for the accurate measurement of parameters such as stain, noise, and pressure [7,8,9,10]. Especially in high-temperature environments, fiber optic F-P pressure sensors show great measurement potential and application prospects [11]. Depending on the structural design, high-temperature F-P pressure sensors are mainly divided into two types: diaphragm-type and open-type sensors. On the one hand, diaphragm-type sensors realize the quantitative detection of pressure by measuring the elastic deformation of the diaphragm under pressure [12,13]. Open-type sensors, on the other hand, measure pressure indirectly through changes in the refractive index of the gas, and its pressure measurement is mainly obtained by calculating the change in optical cavity length (equivalent optical path difference) caused by the change in gas refraction in the F-P cavity [14]. While existing technology has achieved static pressure measurements in environments up to 800 °C [15], most demodulation methods still rely on spectrometers, which limits their application in acquiring high-frequency pressure signals [16,17]. The development of fast spectrometers has led to the availability of devices with a scanning frequency of 30 kHz; however, challenges persist in managing large data volumes at the algorithmic level [18]. Therefore, more suitable algorithms need to be identified for high-speed demodulation systems for pressure sensors.

At present, intensity demodulation serves as a prevalent technique for dynamic signal processing in fiber optic sensors, relying on the light intensity received at the sensor output’s operating point [19]. This method is valued for its system simplicity and rapid response. However, its demodulation range is somewhat restricted and vulnerable to external environmental fluctuations and light-source instability. To address the stability issues associated with intensity demodulation, researchers have proposed a dual-wavelength phase demodulation method. This approach involves utilizing two lasers with a specific wavelength difference to generate a pair of phase signals. The arctan algorithm is then employed to demodulate the signal of the sensor cavity length. This method significantly enhances demodulation stability [20]. However, the dual-wavelength demodulation method poses technical challenges in implementation, particularly in maintaining a consistent and orthogonal-phase difference between the two laser interference signals. Achieving this demands extremely precise matching between the sensor cavity length and the central wavelengths of the lasers [21]. To overcome the stringent requirement of maintaining constant phase differences, some researchers have proposed a dual-wavelength direct current (DC) compensation method. This approach utilizes an algorithm to generate two orthogonal signals that effectively cancel out the DC component in the sensor’s dynamic signal [22]. Other researchers have explored three-wavelength demodulation methods to enable demodulation at arbitrary cavity lengths. For instance, Jiang et al. utilized a self-compensation three-wavelength demodulation method to achieve precise dynamic demodulation [23]. Jia et al. employed a three-wavelength demodulation method combined with an inverse tangent and phase expansion algorithm for demodulating high-frequency signals with arbitrary cavity lengths [24]. Jia et al. employed a three-wavelength passive demodulation algorithm to eliminate the DC component of the sensor’s interferometric signal and mitigate the impact of environmental interference [25]. Therefore, the three-wavelength demodulation technique effectively mitigates phase noise due to fiber bending, offering high frequency, a wide dynamic range, and heightened sensitivity. However, whether the three-wavelength system can be employed for both the dynamic and static demodulation of high-temperature F-P pressure sensors remains a significant challenge.

In this study, a self-compensation three-wavelength demodulation system was developed for demodulating the optical cavity length signals of F-P sensors. Concurrently, a high-temperature pressure sensor was successfully fabricated using the fusion technique and femtosecond laser machining technology. The pressure sensor, along with a thermocouple, was integrated into the three-wavelength demodulation system to form a complete sensing system. Performance tests were conducted under high-temperature, high-pressure, and high-dynamic environments to validate the sensing system’s performance.

## 2. Materials and Methods

**Figure 1:**
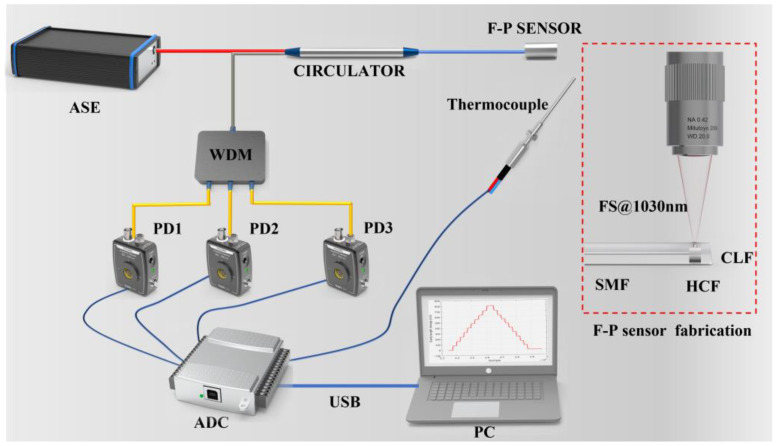
Three-wavelength demodulation and an F-P sensor constitute the sensing system.

The three-wavelength demodulation system and the preparation of the sensor used in this study are shown in Figure 1. The light source of the demodulation system is an amplified spontaneous emission (ASE, Golight, Shenzhen, China) light source, emitting wavelengths from 1528 nm to 1603 nm, with a stable output power of 20 mW. The light emission from the ASE source travels through a circulator and is directed towards the sensor. In the test environment, the pressure signal modulates the interferometric signal reflected by the sensor. The sensor interference signal passes through the circulator (central wavelength 1550 nm) and then enters a 1 × 3 WDM (wavelength division multiplexing, $\lambda_{1}=1553.33\;\mathrm{nm}$, $\lambda_{2}=1557.36\;\mathrm{nm},$ and $\lambda_{3}=1562.23\;\mathrm{nm}$, Flyin, Shenzhen, China). The WDM separates the sensor’s interferometric optical signal into three distinct wavelength channels. The separated light is directed into photodetectors (PDs, Thorlabs, PDA50B2, Newton, NJ, USA), where it is converted from optical signals to electrical signals. The thermocouple (type K, WZDC, Beijing, China) signals for temperature detection and the electrical signals of the PDs are captured by a high-speed data acquisition card (AD7606, ANALOG DEVICES, Wilmington, MA, USA). This data acquisition card provides a maximum sampling rate of 200 kHz. These data are then transferred to the host computer via a universal serial bus (USB). The software filters the acquired voltage signals and applies a three-wavelength demodulation algorithm to process the signals.

The structure of the sensor fabricated in this experiment is single-mode fiber–hollow-core fiber–coreless fiber (SMF-HCF-CLF). The sensor is prepared by fiber optic fusion technology, fiber optic polishing technology, and femtosecond laser machining technology. The specific preparation process involved several steps: Initially, SMF (G652, YOFC, Wuhan, China) and HCF (inner diameter 75 µm, Ifiber, Ningbo, China) were end-faceted using a fiber cutter. Subsequently, a fiber fusion splicer (S178, FITEL, Chiba, Japan) was employed to fuse the SMF to the HCF. A fiber optic cutter with a microscope was used to cut the appropriate cavity length. The same fusion method was applied to connect the CLF (YOCF, China) to the opposite end of the HCF. Thirdly, a femtosecond laser (wavelength 1030 nm, power 10 W, repetition frequency 200 kHz, WOP, Vilnius, Lithuania) was utilized to process the gas channel within the HCF. Finally, an 8° bevel was polished on the other end of the CLF to improve spectral quality by reducing non-essential light reflections. This sensor design is characterized by its simplicity, compact size, and ease of integration.

The sensor prepared in this paper forms an F-P interferometer inside an HCF. The reflectivity of the F-P cavity of the sensor is only 4%, forming a low-fine FP cavity. Its two-beam interference signal is shown as follows [26]:

(1) $$I_{R}=A+Bcos(\frac{4n\pi L_{0}}{\lambda_{i}}),(i=1,2,3)$$

where $I_{R}$ is the reflected light intensity of the sensor. A is the DC component of the interferometric signal of the sensor. B is the amplitude. $\lambda_{i}\;(i=1,2,3)$ is the central wavelength of the three interference signals separated by WDM. n is the effective refractive index of the F-P cavity of the sensor, which is approximated as 1 for air. $L_{0}$ is the length of the F-P cavity of the sensor.

According to Equation (1), in the initial static state of the FP cavity, the light intensity signal detected by the photodetectors can be expressed as follows:

(2) $$\left\{\begin{array}{c} I_{1}=A+B\cos(\theta_{1}),\theta_{1}=\frac{4n\pi L_{0}}{\lambda_{1}}\\ I_{2}=A+B\cos(\theta_{2}),\theta_{2}=\frac{4n\pi L_{0}}{\lambda_{2}}\\ I_{3}=A+B\cos(\theta_{3}),\theta_{3}=\frac{4n\pi L_{0}}{\lambda_{3}} \end{array}\right.$$

where $I_{1}$, $I_{2},$ and $I_{3}$ can be replaced by the light intensity signals detected by PD1, PD2, and PD3, respectively. $\theta_{1}$, $\theta_{2}$, and $\theta_{3}$ are the initial phases that are calculated at the beginning of the algorithm.

At the beginning of the test, the cavity length of the sensor starts to change. The phase acquired by the PDs changes, and the intensity of the light it detects can be expressed as follows:

(3) $$\left\{\begin{array}{c} I_{1}=A+B\cos(\theta_{1}+\Delta \theta_{1}),\Delta \theta_{1}=\frac{4n\pi (\Delta L)}{\lambda_{1}}\\ I_{2}=A+B\cos(\theta_{2}+\Delta \theta_{2}),\Delta \theta_{2}=\frac{4n\pi (\Delta L)}{\lambda_{2}}\\ I_{3}=A+B\cos(\theta_{3}+\Delta \theta_{3}),\Delta \theta_{3}=\frac{4n\pi (\Delta L)}{\lambda_{3}} \end{array}\right.$$

where ΔL is much smaller than $L_{0}$, and $\lambda_{1},$ $\lambda_{2},$ and $\lambda_{3}$ are similar, so $\Delta \theta_{1}$, $\Delta \theta_{2}$, and $\Delta \theta_{3}$ can be set as equal. The phase of the transform in the three optical paths is replaced by Δθ. In this case, the detected light intensity can be expressed as follows:

(4) $$\left\{\begin{array}{c} I_{1}=A+B\cos(\theta_{1}+\Delta \theta )\\ I_{2}=A+B\cos(\theta_{2}+\Delta \theta )\\ I_{3}=A+B\cos(\theta_{3}+\Delta \theta ) \end{array}\right.$$

expanding Equation (4) as follows:

(5) $$\left\{\begin{array}{c} I_{1}=A+B\cos(\theta_{1})\cos(\Delta \theta )-B\sin(\theta_{1})\sin(\Delta \theta )\\ I_{2}=A+B\cos(\theta_{2})\cos(\Delta \theta )-B\sin(\theta_{2})\sin(\Delta \theta )\\ I_{3}=A+B\cos(\theta_{3})\cos(\Delta \theta )-B\sin(\theta_{3})\sin(\Delta \theta ) \end{array}\right.$$

To remove the DC component, the following is calculated: $I_{1}-I_{2}$, $I_{3}-I_{2}$. $\theta_{1}$, $\theta_{2}$, and $\theta_{3}$ are the initial phases and are computed at the beginning of the algorithm. Thus, the quadrature signal of the phase variation can be expressed as follows:

(6) $$\left\{\begin{array}{c} F_{1}=B\sin(\Delta \theta )=\frac{\left(f_{1}-f_{2})(cos\theta_{2}-cos\theta_{3})-(f_{2}-f_{3})(cos\theta_{1}-cos\theta_{2}\right)}{\left(cos\theta_{1}-cos\theta_{2})(sin\theta_{2}-sin\theta_{3})-(cos\theta_{2}-cos\theta_{3})(sin\theta_{1}-sin\theta_{2}\right)}\\ F_{2}=B\cos(\Delta \theta )=\frac{\left(f_{1}-f_{2})(sin\theta_{2}-sin\theta_{3})-(f_{2}-f_{3})(sin\theta_{1}-sin\theta_{2}\right)}{\left(cos\theta_{1}-cos\theta_{2})(sin\theta_{2}-sin\theta_{3})-(cos\theta_{2}-cos\theta_{3})(sin\theta_{1}-sin\theta_{2}\right)} \end{array}\right.$$

The algorithm removes the DC component of the interference spectrum, as seen in the computational equation in Equation (6). Two orthogonal signals are obtained, which are solved by the arctan algorithm for Δθ, the expression of which is as follows:

(7) $$\Delta \theta =arctan\frac{F_{1}}{F_{2}}$$

The final amount of variation in the cavity length ΔL obtained can be expressed as follows:

(8) $$\Delta L=\frac{\lambda_{2}}{4n\pi }\Delta \theta$$

**Figure 2:**
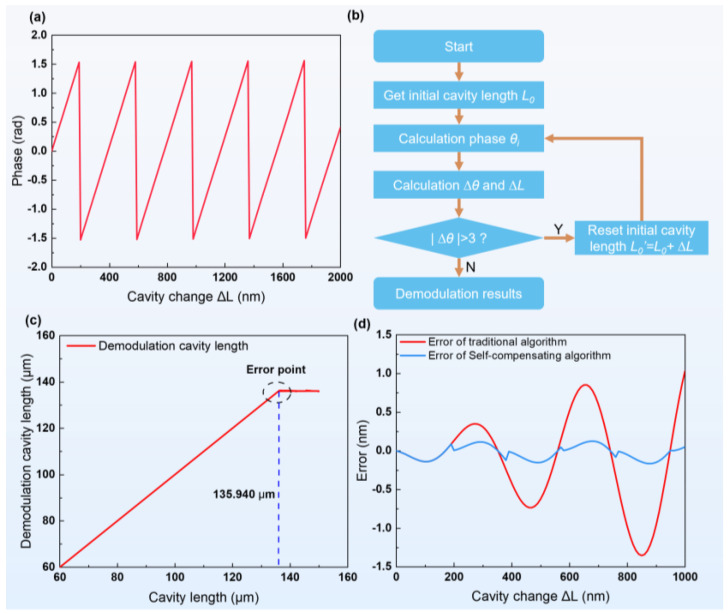
(**a**) Demodulation of the changed cavity length in relation to the phase. (**b**) Self-compensating three-wavelength algorithmic flow. (**c**) Demodulation error points. (**d**) Comparison of the demodulation error of the self-compensating algorithm and the conventional algorithm.

Because the value range of the arctan algorithm is in (−π/2, π/2), as shown in Figure 2a if the Δθ demodulation range exceeds this range, demodulation will jump and eventually lead to an increase in the error of the demodulation result. To solve this problem, a novel phase compensation method is proposed in this paper for identifying and compensating the phase jump points. An accurate phase value Δθ′ is also obtained. This demodulation method is shown in Figure 2b.

The demodulation method consists of the following steps:

1. The dynamic sampling point calculation, when $\left|\Delta \theta \right|\geq 3$, can be shown in Figure 2a, which is the moment of the occurrence of phase jump. The setting of the phase threshold is related to the sampling rate; the higher the sampling rate, the closer the threshold is to π. In this paper, we experimentally measured whether setting the threshold to 3 can adapt to the phase mutation problem at the sampling rate of 100 Hz~200 kHz.
2. Compensate for the change in lumen length, calculated before the jump to the initial lumen length as $L_{0}\prime =L_{0}+\Delta L$.
3. Recalculate the initial phase according to Equation (3).
4. Recalculate Equations (6)–(8) at the jump point to obtain the compensated cavity length value as $L\prime =L_{0}\prime +\Delta L\prime$.

This demodulation method constantly corrects the initial phase, which effectively solves the problem of sudden phase change. At the same time, the demodulation error does not accumulate as the cavity length increases.

To verify the applicability of the F-P cavity length and assess the demodulation accuracy of the algorithm, simulations were conducted. Reasonable selection of the central wavelength is required when selecting the WDM. When the phase difference between any two signals in the initial phase $\theta_{1}$, $\theta_{2},$ and $\theta_{3}$ is close to 2π, one signal can be covered by the other. We selected the standard WDM signal near 1550 nm for analysis, as $\lambda_{1}=1553.33\;nm$, $\lambda_{2}=1557.36\;nm,$ and $\lambda_{3}=1562.23\;nm$. We performed the simulation using MATLAB R2019a. The initial cavity length was set to 60 µm, and the simulation results are shown in Figure 2c, which shows the mutation point at 135.94 µm. As shown in Figure 2d, the cavity length error, after demodulation and after the compensation algorithm, is simulated at the sensor operating range of 1000 nm. The simulation results show that the error is controlled within ±0.2 nm in the operating range of the sensor.

## 3. Results

### 3.1. Construction of a Sensor Static Pressure Test Setup

**Figure 3:**
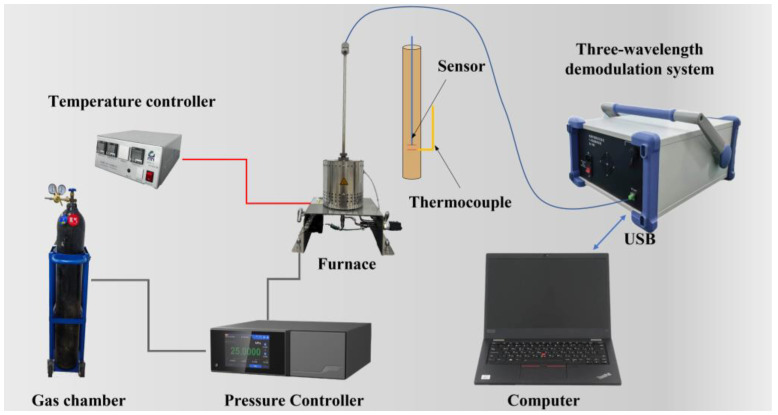
Static pressure and high-temperature pressure test system.

To fully evaluate the performance of the proposed sensing system, static high-temperature pressure testing, and dynamic testing experiments were designed and implemented in this study. For static testing, an experimental system was used, as shown in Figure 3. This system uses a furnace to create the required high-temperature environment and a nitrogen through-pressure controller (Const860, Const, Beijing, China) to deliver a steady pressure of nitrogen to the furnace. The temperature inside the furnace is tightly controlled by a precise temperature controller to ensure consistent test conditions. The sensor is placed at the upper end of the furnace to simulate the high-temperature environment of the actual application. The signals from the sensors and thermocouples are acquired and processed by a three-wavelength demodulation system, the inner workings of which are shown in Figure 1. The demodulation system accurately captures changes in the optical cavity length (equivalent optical path difference: OPD) of the sensors and converts these changes into readable pressure data. Ultimately, these measurements are transmitted to a host computer for display and further analysis.

### 3.2. Room Temperature and Static Pressure Test Results

**Figure 4:**
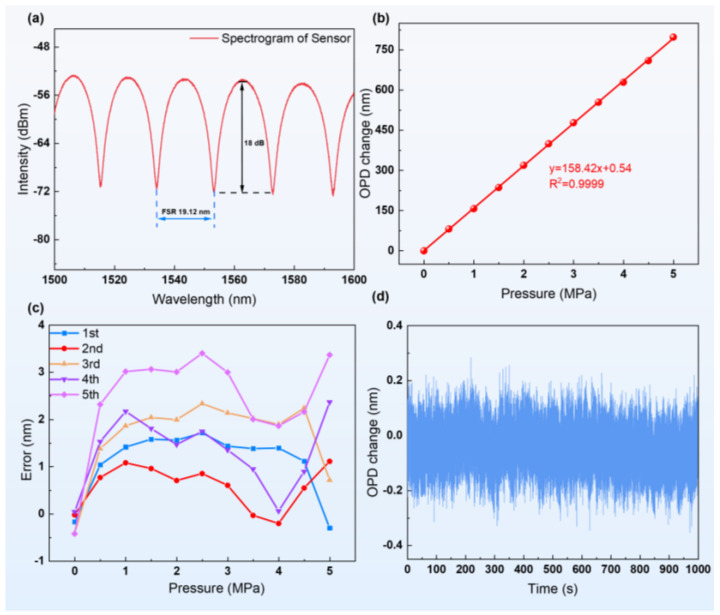
(**a**) Spectrogram of the proposed sensor. (**b**) The linear relationship between the OPD and the pressure of the sensor at room temperature. (**c**) Multiple test errors. (**d**) Sensing-system static noise.

The experiment verifies the output characteristics of the sensing system at room temperature. Figure 4a shows the spectrogram of the prepared sensor, indicating an excellent spectral output with a free spectral range (FSR) of about 19.12 nm and an interference fringe contrast of about 18 dB. The cavity length of the sensor in the initial state $L_{0}=60,611\;nm$ was obtained by the white-light interference (WLI) method [27].

The experiment involved conducting static pressure tests using a pressure controller, ranging from 0 to 5 MPa in increments of 0.5 MPa. The OPD value was recorded after stabilizing for 15 s at each step. The test results demonstrated the exceptional linearity (R^2^ = 0.9999) of the sensor, confirming its accuracy and reliability in pressure measurements, as shown in Figure 4b. The slope of the measured data determined the pressure sensitivity of the sensor, calculated as *Sp* = 158.42 nm/MPa. Based on the gas refractive index n–pressure relationship, we derived the following pressure theoretical sensitivity equation [28]:

(9) $$S_{p}=\frac{dOPD}{dP}=\frac{dn}{dP}L_{0}=\frac{2.8793\times 10^{-3}}{1+0.003661\times T}L_{0}$$

where the initial cavity length of the sensor $L_{0}$ is 60,611 nm, and the test temperature is 25 °C in a room temperature environment. The theoretical calculation result was 159.88 nm/MPa. The experimental results show that the actual measured values are very close to the theoretical predictions, thus verifying the high demodulation accuracy of the sensing system. These results not only confirm the validity of the sensor design but also demonstrate the high sensitivity and accuracy of the three-wavelength demodulation system for static pressure measurements.

The repeatability and stability of the sensor were also evaluated in this study. Five pressure tests were executed at room temperature, and the values at each pressure point were recorded and compared with the linearity measurements to assess the repeatability error of the sensor. As shown in Figure 4c, the maximum deviation in the five rounds of testing was only 3.6 nm, indicating the excellent repeatability of the sensor. Further, to explore the static stability and test the noise of the demodulation system, we continuously acquired data for 1000 s at a sampling rate of 100 Hz. As shown in Figure 4d, the sensor and demodulation system show good long-term stability, and the variations in OPD are controlled within ±0.2 nm.

### 3.3. High-Temperature Static Pressure Test Results

**Figure 5:**
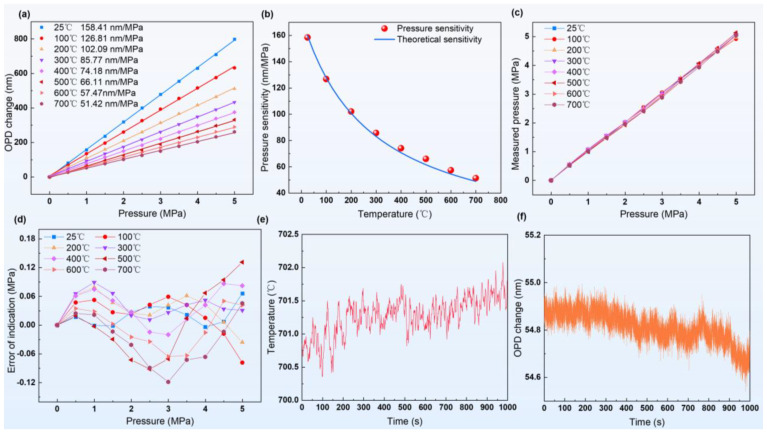
(**a**) Relationship between pressure and OPD at different temperatures. (**b**) Measured pressure sensitivity versus theoretical value error. (**c**) Measured pressure values versus standard pressure. (**d**) Measured pressure error at various temperatures and pressure test points. (**e**,**f**) Stability of thermocouple measurement and OPD at 700 °C.

In this study, the performance characteristics of the two-parameter demodulation of the thermocouple temperature and F-P cavity length were tested. Based on the static pressure response test at room temperature, the experiment was extended to the high-temperature interval from 100 °C to 700 °C, with every 100 °C as a test node, and the pressure response of the sensor at different temperatures was evaluated. The experiments began by gradually increasing the temperature in the furnace to a preset target and keeping it stable for half an hour to ensure thermal stability. Subsequently, nitrogen was introduced at each pressure test point for regulation and was maintained for 30 s before recording the OPD data. By normalizing the pressure baseline data, the pressure-versus-temperature response was established over the range of 25 °C to 700 °C, as shown in Figure 5a. The test results showed that the sensor exhibited high linearity at all temperature points, verifying its ability to measure accurately in high-temperature environments.

The sensor’s sensitivity at various temperatures was compared with theoretical Equation (9) data. As shown in Figure 5b, the results revealed a deviation between experimentally measured sensitivity and theoretical values at higher temperatures attributed to signal attenuation in high-temperature environments. To address this challenge, we employed an integrated fitting method that combined thermocouple temperature data collected by the demodulation system with OPD demodulation data. This approach effectively ensured accurate pressure signal output across the entire temperature range from 25 °C to 700 °C, thereby enhancing sensor measurement accuracy under high-temperature conditions [12]. The fitting equation is as follows:

(10) $$P=\frac{1}{S_{p}}\times \Delta L=\frac{1+0.00327\times T}{2.81\times 10^{-3}\times L_{0}}\Delta L$$

where *P* is the output pressure value, *T* is the thermocouple temperature data collected by the demodulation system, $L_{0}$ is the initial cavity length of the sensor, and ΔL is the measured OPD value. The pressure output result is shown in Figure 5c. The error in the indicated value of the pressure measured by the sensor at each temperature point is shown in Figure 5d. The maximum error was about −0.13 MPa. After the high-temperature and high-pressure tests, we tested the stability of the thermocouple and the demodulated OPD. The thermocouple part was set with a sampling rate of 1 Hz, the OPD was sampled at 100 Hz, and the test duration was 1000 s. The test results are shown in Figure 5e,f. Due to the systematic error of the furnace, the maximum change in the measured temperature test result was 1.5 °C. The maximum variation in the measured OPD result was 0.5 nm. This proves that the sensor has good high-temperature stability, and the demodulation system has high stability.

### 3.4. Dynamic Pressure Test Results

**Figure 6:**
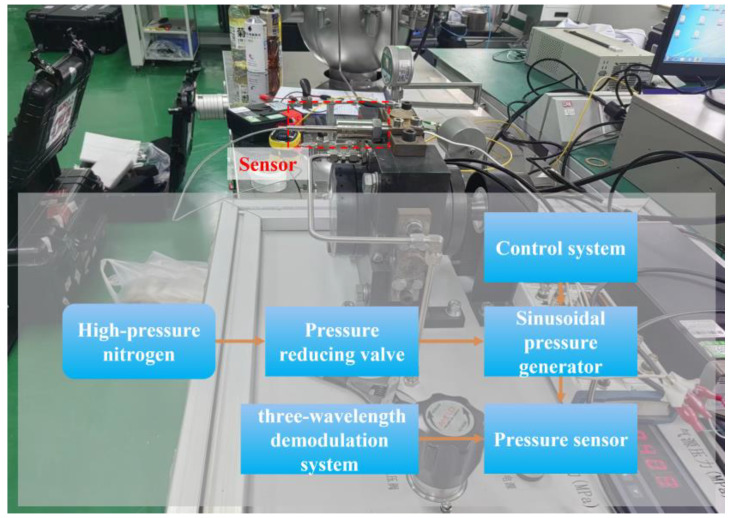
Sinusoidal pressure generator system.

To investigate the response of the sensor and demodulation system to high-frequency pressure signals, the sensor was tested with a sinusoidal pressure generator. This generator is specifically designed to produce periodic variations in pressure, creating a sinusoidal waveform that quickly enters the test cavity to simulate dynamic pressure conditions typical of real-world applications. The test setup, depicted in Figure 6, comprises a sinusoidal pressure generator, sensor, demodulation system, control system, and other necessary types of auxiliary equipment. The sinusoidal pressure generator precisely controls the air pressure input to dynamically load the sensor. The sensor converts the received pressure signal into an optical signal, which is subsequently captured and processed by the demodulation system.

**Figure 7:**
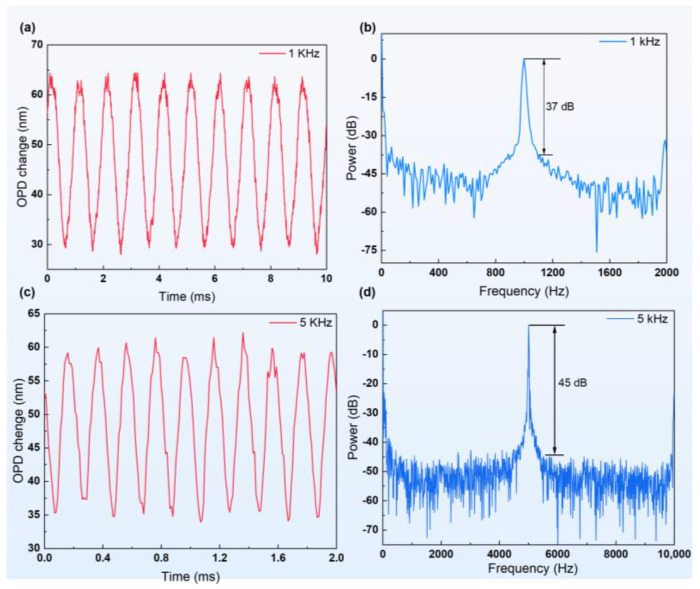
(**a**,**c**) 1 kHz and 5 kHz sinusoidal pressure time domain signals, respectively. (**b**,**d**) are the frequency domain signals corresponding to 1 kHz and 5 kHz, respectively.

The demodulation system operated at a sampling rate of 100 kHz, and the sensor’s output was tested under sinusoidal pressure frequencies of 1000 Hz and 5000 Hz. The sensor exhibited frequency-dependent responses at both frequencies. For the 1000 Hz sinusoidal pressure test, the sensor showed an amplitude of approximately 35 nm, as depicted in Figure 7 a. The test’s resonant frequency was 1000 Hz, achieving an SNR of over 37 dB, as shown in Figure 7b. The demodulation system was configured similarly to the test conditions.

During the 5000 Hz sinusoidal pressure test, the sensor exhibited a reduced amplitude of 25 nm, illustrated in Figure 7c. This decrease is attributed to the higher operational frequency of the sinusoidal pressure system coupled with lower inlet pressures. The test’s characteristic frequency was 5000 Hz, with SNR exceeding 45 dB, as depicted in Figure 7d. These high-frequency experiments confirm that the sensing system proposed in this study exhibits robust dynamic response characteristics suitable for high-frequency pressure environments.

## 4. Discussion

The sensing system proposed in this paper shows excellent overall performance under high-temperature, high-pressure, and high-dynamic environments. The sensing system is experimentally verified to be able to accurately measure the pressure changes in the sensor under static and dynamic conditions, showing good linearity, sensitivity, and accuracy.

In static testing, the open-cavity pressure sensor was able to maintain a high linear pressure response over the range of 25 °C to 700 °C, demonstrating the stability and reliability of this type of sensor in high-temperature environments. Although the experimental sensitivity was in error from the theoretical value as the temperature increased, the fitting of the thermocouple data collected by the demodulation system to the OPD demodulation data was able to obtain a pressure signal output over the full temperature range with a maximum indication error of 0.13 MPa.

The dynamic test results show that the sensor and the demodulation system can accurately reflect the pressure changes at sinusoidal pressure frequencies of 1000 Hz and 5000 Hz and display good dynamic response characteristics. Especially in the high-frequency test at 5000 Hz, the sensor still maintained a high SNR, which was higher than 45 dB, proving that this type of sensing system has a wide range of potential applications in high-frequency pressure change environments.

The above test results show that the three-wavelength demodulation algorithm proposed in this paper effectively solves the phase jump problem through the adaptive phase compensation method and improves the accuracy and stability of the measurement. The demodulation system has excellent static and dynamic acquisition functions, and the demodulation error is effectively controlled within ±0.1 nm.

Table 1 provides a comprehensive summary of recent research on high-speed, high-frequency demodulation systems for F-P sensors. The table details the progress made by different research teams on high-speed demodulation techniques for FP sensors, including the demodulation method, maximum sampling rate, the range of cavity lengths that can be applied, and on which sensors validation has been performed. The use of the three-wavelength self-compensation algorithm proposed in this paper significantly improved the stability and reliability of the measurement while ensuring high-speed demodulation. It was tested in static, dynamic, high-temperature, and high-pressure environments.

## 5. Conclusions

In conclusion, a self-compensating three-wavelength demodulation system was successfully developed and integrated with an F-P pressure sensor and a thermocouple to construct a comprehensive sensing system. The system exhibited excellent dynamic and static stability in pressure tests. The accuracy of the proposed demodulation system in terms of precise demodulation was verified by room-temperature static experiments. Further high-temperature and high-pressure experiments not only confirmed the measurement capability of the sensor under extreme temperature conditions but also demonstrated the excellent performance of the demodulation system in terms of static temperature–pressure decoupling. Dynamic experiments further demonstrated the sensor system’s ability to accurately capture sinusoidal pressure variations up to 5 kHz, and its performance was verified through high-frequency testing. The sensing system had a noise level of 0.4 nm under static conditions and a resolution of 0.5 nm, and it supported sampling frequencies up to 200 kHz. The system demonstrated high accuracy, a wide measurement range, and high resolution in fiber optic pressure demodulation. In addition, the system has a simple and compact design, with great potential for high integration and wide application in engineering applications. These features give the sensor system proposed in this study a significant competitive advantage in the field of high-precision pressure measurement and provide an important experimental basis and theoretical support for technological progress in related fields.

## Figures and Tables

**Table 1:** Some studies on high-speed demodulation methods for F-P sensors.

Demodulation Method	Demodulation Speed	Demodulation FP Cavity Length	Verify the Type of Sensor
High-speed spectral demodulation based on scanning lasers [18]	2 kHz	300–130,000 μm	Vibration verification of piezoelectric ceramic
Three-wavelength phase demodulation method [23]	20 kHz	23.065–1094.703 μm	Vibration verification of piezoelectric ceramic
Self-compensation three-wavelength demodulation method [24]	500 kHz	44.885–83.347 μm	FP pressure sensor
Dynamic phase white-light interferometry [29]	20 kHz	Not explicitly mentioned	FP vibration sensor
Demodulation based on dual Fizeau interferometer [30]	100 kHz	4.74 μm (range of variation)	FP vibration sensor
Phase demodulation method for high-speed spectrometers [31]	20 kHz	Not explicitly mentioned	FP vibration/acoustic sensor
High-speed demodulation based on coarse spectrum [32]	50 kHz	25.66–684.13 μm	Vibration verification of piezoelectric ceramic
High-speed spectral peak-jump compensation demodulation [33]	5 kHz	Not explicitly mentioned	FP high-temperature pressure sensor
This study	200 kHz	60–135 um	FP high-temperature pressure sensor

## Data Availability

The data presented in this study are available on request from the corresponding author.

## References

[1] Chen Y., Xu Q., Zhang X., Kuang M. (2023). Materials and Sensing Mechanisms for High-Temperature Pressure Sensors: A Review. IEEE Sens. J..

[2] Yao Z., Liang T., Jia P., Hong Y., Qi L., Lei C., Zhang B., Xiong J. (2016). A High-Temperature Piezoresistive Pressure Sensor with an Integrated Signal-Conditioning Circuit. Sensors.

[3] Ren J., Ward M., Kinnell P., Craddock R., Wei X. (2016). Plastic Deformation of Micromachined Silicon Diaphragms with a Sealed Cavity at High Temperatures. Sensors.

[4] Chen L., Tian J., Wu Q., Li J., Yao Y., Wang J. (2023). Temperature-Insensitive Gas Pressure Sensor Based on Photonic Crystal Fiber Interferometer. IEEE Sens. J..

[5] Zhang Y., Jiang Y., Deng H., Gao H., Tang C., Wang X. (2023). All-sapphire-based optical fiber pressure sensor with an ultra-wide pressure range based on femtosecond laser micromachining and direct bonding. Opt. Express.

[6] Shao M., Cao Z., Gao H., Yu D., Qiao X. (2023). Optical fiber ultrasonic sensor based on partial filling PDMS in hollow-core fiber. Opt. Laser Technol..

[7] Fan H., Zhang L., Gao S., Chen L., Bao X. (2019). Ultrasound sensing based on an in-fiber dual-cavity Fabry–Perot interferometer. Opt. Lett..

[8] Wong K.P., Kim H.-T., Rajasekaran K., Yazdkhasti A., Sai Sudhakar B., Wang A., Lee S.E., Kiger K., Duncan J.H., Yu M. (2022). High-speed, large dynamic range spectral domain interrogation of fiber-optic Fabry–Perot interferometric sensors. Appl. Opt..

[9] Guo T., Zhang T., Qiao X. (2020). FBG-EFPI sensor for large strain measurement with low temperature crosstalk. Opt. Commun..

[10] Zhang J., Zhao X., Zheng Y., Chen J., Bai J., Wu L., Gao X., Li Z., Xue C. (2023). A Wide Frequency Response Fabry–Pérot Acoustic Sensor Based on the Self-Stabilization System. IEEE Sens. J..

[11] Hu X., Su D., Qiao X. (2024). Highly sensitive optical fiber pressure sensor based on the FPI and Vernier effect via femtosecond laser plane-by-plane writing technology. Appl. Opt..

[12] Zhang Y., Jiang Y. (2023). An All-Sapphire Fiber Diaphragm-Free Extrinsic Fabry–Perot Interferometric Sensor for the Measurement of Gas Pressure at Ultrahigh Temperature. IEEE Sens. J..

[13] Jian T., Guolu Y., Changrui L., Shen L., Zhengyong L., Xiaoyong Z., Qiao W., Jing Z., Kaiming Y., Yiping W. (2015). High-Sensitivity Gas Pressure Sensor Based on Fabry–Pérot Interferometer with a Side-Opened Channel in Hollow-Core Photonic Bandgap Fiber. IEEE Photonics J..

[14] Li Z., Tian J., Jiao Y., Sun Y., Yao Y. (2019). Simultaneous Measurement of Air Pressure and Temperature Using Fiber-Optic Cascaded Fabry–Perot Interferometer. IEEE Photonics J..

[15] Ma W., Jiang Y., Gao H. (2019). Miniature all-fiber extrinsic Fabry–Pérot interferometric sensor for high-pressure sensing under high-temperature conditions. Meas. Sci. Technol..

[16] Liu W., Yang T., Shi Y., Wu J., Dong Y. (2023). Combined Interrogation Algorithm of Phase Function and Minimum Mean Square Error for Fiber-Optic Fabry-Perot Micro-Pressure Sensors Based on White Light Interference. J. Light. Technol..

[17] Wei X., Yan H., Liao N., Ma L., He L., Hou H., Wang W. (2022). A visible-light spectral demodulation system for fiber-optic SiC Fabry-Perot sensors. Optik.

[18] Xu Y., Qi H., Zhao X., Li C., Chen K. (2024). High-speed spectrum demodulation of fiber-optic Fabry–Perot sensor based on scanning laser. Opt. Lasers Eng..

[19] Ximin Z., Sen Q., Huixin L., Chuan C., Chuanlu D., Chengyong H., Yi H. (2023). An extrinsic Fabry–Pérot interference fiber sensorfor ultrasonic detection of partial discharge. Opt. Appl..

[20] Liao H., Lu P., Liu L., Wang S., Ni W., Fu X., Liu D., Zhang J. (2017). Phase Demodulation of Short-Cavity Fabry–Perot Interferometric Acoustic Sensors with Two Wavelengths. IEEE Photonics J..

[21] Jia J., Jiang Y., Zhang L., Gao H., Wang S., Jiang L. (2018). Dual-Wavelength DC Compensation Technique for the Demodulation of EFPI Fiber Sensors. IEEE Photonics Technol. Lett..

[22] Ren Q., Jia P., An G., Liu J., Fang G., Liu W., Xiong J. (2021). Dual-wavelength demodulation technique for interrogating a shortest cavity in multi-cavity fiber-optic Fabry–Pérot sensors. Opt. Express.

[23] Jia J., Jiang Y., Cui Y. (2020). Phase Demodulator for the Measurement of Extrinsic Fabry-Perot Interferometric Sensors With Arbitrary Initial Cavity Length. IEEE Sens. J..

[24] Ren Q., Jia P., An G., Liu J., Liu W., Xiong J. (2023). Self-compensation three-wavelength demodulation method for the large phase extraction of extrinsic Fabry–Pérot interferometric sensors. Opt. Lasers Eng..

[25] Jia J., Jiang Y., Gao H., Zhang L., Jiang Y. (2019). Three-wavelength passive demodulation technique for the interrogation of EFPI sensors with arbitrary cavity length. Opt. Express.

[26] Chen P., Dai Y., Zhang D., Wen X., Yang M. (2018). Cascaded-Cavity Fabry-Perot Interferometric Gas Pressure Sensor based on Vernier Effect. Sensors.

[27] Zhou X., Yu Q. (2011). Wide-Range Displacement Sensor Based on Fiber-Optic Fabry–Perot Interferometer for Subnanometer Measurement. IEEE Sens. J..

[28] Zhang Y., Jiang Y., Yang S., Zhang D. (2024). All-sapphire fiber-optic sensor for the simultaneous measurement of ultra-high temperature and high pressure. Opt. Express.

[29] Liu Q., Jing Z., Liu Y., Li A., Xia Z., Peng W. (2021). Absolute Measurement of Dynamic Low-Finesse Fabry–Perot Cavity Using Phase-Shifting White-Light Interferometry. J. Light. Technol..

[30] Kong D., Song Z., Wang N., Wang Z., Huang P., Zhu Y., Zhang J. (2022). Fiber Fabry-Perot Demodulation System Based on Dual Fizeau Interferometers. Photonic Sens..

[31] Li C., Zhao X., Qi H., Wang Z., Xu Y., Han X., Huang J., Guo M., Chen K. (2024). Integrated fiber-optic Fabry–Perot vibration/acoustic sensing system based on high-speed phase demodulation. Opt. Laser Technol..

[32] Zhang P., Wang Y., Chen Y., Lei X., Qi Y., Feng J., Liu X. (2021). A High-Speed Demodulation Technology of Fiber Optic Extrinsic Fabry-Perot Interferometric Sensor Based on Coarse Spectrum. Sensors.

[33] Li Z., Wang S., Shao Z., Jiang J., Yan M., Sun Z., Yang H., Dai X., Liu T. (2024). High-Speed and Error-Suppressed Fiber-Optic F–P System for Dynamic Pressure Measurement at High Temperature. IEEE Sens. J..

